# MRI-Negative Transverse Myelitis Revealing Seropositive Rheumatoid Arthritis in a Young Woman

**DOI:** 10.7759/cureus.89330

**Published:** 2025-08-04

**Authors:** Valentina Roa Forster, Lisett Castellanos, Mohammed Khatib, Gabriela Perez

**Affiliations:** 1 Neurology, Herbert Wertheim College of Medicine, Florida International University, Miami, USA; 2 Neurology, Palmetto General Hospital, Miami, USA

**Keywords:** autoimmune neurology, central nervous system inflammation, extra-articular manifestations, intravenous immunoglobulin, mri-negative myelitis, neuroimmunology, rheumatoid arthritis, seropositive ra, transverse myelitis

## Abstract

Transverse myelitis (TM) is an inflammatory disorder of the spinal cord often associated with autoimmune diseases, such as systemic lupus erythematosus (SLE) or neuromyelitis optica spectrum disorder (NMOSD); however, it is rarely linked to rheumatoid arthritis (RA). We present the case of a 28-year-old woman with subacute ascending numbness, lancinating pain, and bilateral lower extremity weakness resulting in significant functional impairment. Despite upper motor neuron signs on examination and supportive cerebrospinal fluid findings, including elevated gamma globulins and positive myelin basic protein, spinal MRI remained negative. The patient failed to improve with corticosteroids but showed a significant response to intravenous immunoglobulin. Electrophysiological studies indicated a central process, and serologic workup later revealed rheumatoid factor positivity. She was ultimately diagnosed with seropositive RA and started on certolizumab pegol. Alternative diagnoses, including multiple sclerosis (MS), NMOSD, Guillain-Barré syndrome, and compressive or ischemic myelopathy, were ruled out. This case highlights the diagnostic challenge of MRI-negative TM and its potential association with systemic autoimmune disease, specifically RA. It emphasizes the importance of clinical vigilance, early cerebrospinal fluid analysis, and prompt immunotherapy even in the absence of imaging abnormalities. Our findings broaden the neurologic spectrum of RA and support the role of infection-triggered immune dysregulation in autoimmune myelitis.

## Introduction

Transverse myelitis (TM) is an inflammatory condition involving immune-mediated mechanisms triggered by autoimmune disorders, infections, or parainfectious agents. Given the broad differential of TM, infectious etiologies such as Lyme disease should also be considered in appropriate clinical and geographic contexts. It is characterized by lymphocyte and monocyte infiltration of the perivascular spaces and segments of the spinal cord, as well as astroglial and microglial activation. In TM, white and gray matter may be equally affected [1]. TM typically presents with sensory disturbance and motor weakness. Patients can also experience autonomic dysfunction, characterized by symptoms such as bladder or bowel issues, defined sensory levels, and upper motor neuron signs, including hyperreflexia, positive Babinski signs, and spasticity. In most cases, magnetic resonance imaging (MRI) reveals T2-hyperintense lesions that primarily affect the cervical area and involve less than one vertebral body. Gadolinium enhancement is observed in more than half of the patients, indicating active inflammation. The absence of imaging findings does not rule out TM. MRI-negative TM occurs in approximately 40% of patients on first imaging, with only 14% of patients showing lesions on subsequent MRIs. In these patients, a diagnosis can be made if cerebrospinal fluid (CSF) analysis demonstrates pleocytosis, elevated protein levels, or an elevated IgG index [2-5].

Autoantibody-mediated neurological diseases can arise from molecular mimicry, immune complex depositions, or superantigen-induced immune activation after infection [6,7]. While TM is commonly associated with autoimmune conditions such as systemic lupus erythematosus (SLE) or neuromyelitis optica spectrum disorder (NMOSD), its occurrence in rheumatoid arthritis (RA) is exceedingly rare. RA is a systemic autoimmune disease that classically affects synovial joints; however, extra-articular manifestations can occur and, although rare, may involve the central nervous system (CNS). RA's pathogenesis involves widespread immune dysregulation, including the production of autoantibodies, cytokine signaling, and the activation of T and B cells. In cases of TM associated with RA, diagnosis is often delayed due to the rarity of this presentation and the lack of overt joint symptoms. Recent case reports documenting an association between RA and NMOSD raise questions about shared autoimmune susceptibility and the possibility of CNS demyelination in RA [8-10].

## Case presentation

We report a 28-year-old female with no significant past medical history except for a prior skin infection treated with long-term antibiotics, likely within the past year, though she could not recall the exact timing. Family history was significant for ankylosing spondylitis in the father. She initially presented to the emergency department (ED) with symmetric numbness in bilateral distal lower extremities that had been progressively ascending over the prior two to three weeks. She had not sought prior medical attention prior to presentation to the ED. The patient’s symptoms were associated with 10/10 lancinating pain that limited her ability to sleep, perform daily activities, and ambulate, requiring a wheelchair. She denied bowel, bladder, or sexual dysfunction. There was no record of recent vaccinations before symptom onset. She reported mild joint stiffness, but denied joint swelling, joint pain, rash, oral ulcers, or constitutional symptoms such as fever or weight loss. Although originally developed for multiple sclerosis, the Expanded Disability Status Scale (EDSS) has been applied to assess functional impairment in patients with transverse myelitis and other demyelinating disorders [2]. In this case, the patient’s EDSS score was greater than five, indicating a significant level of disability.

**Figure 1:**
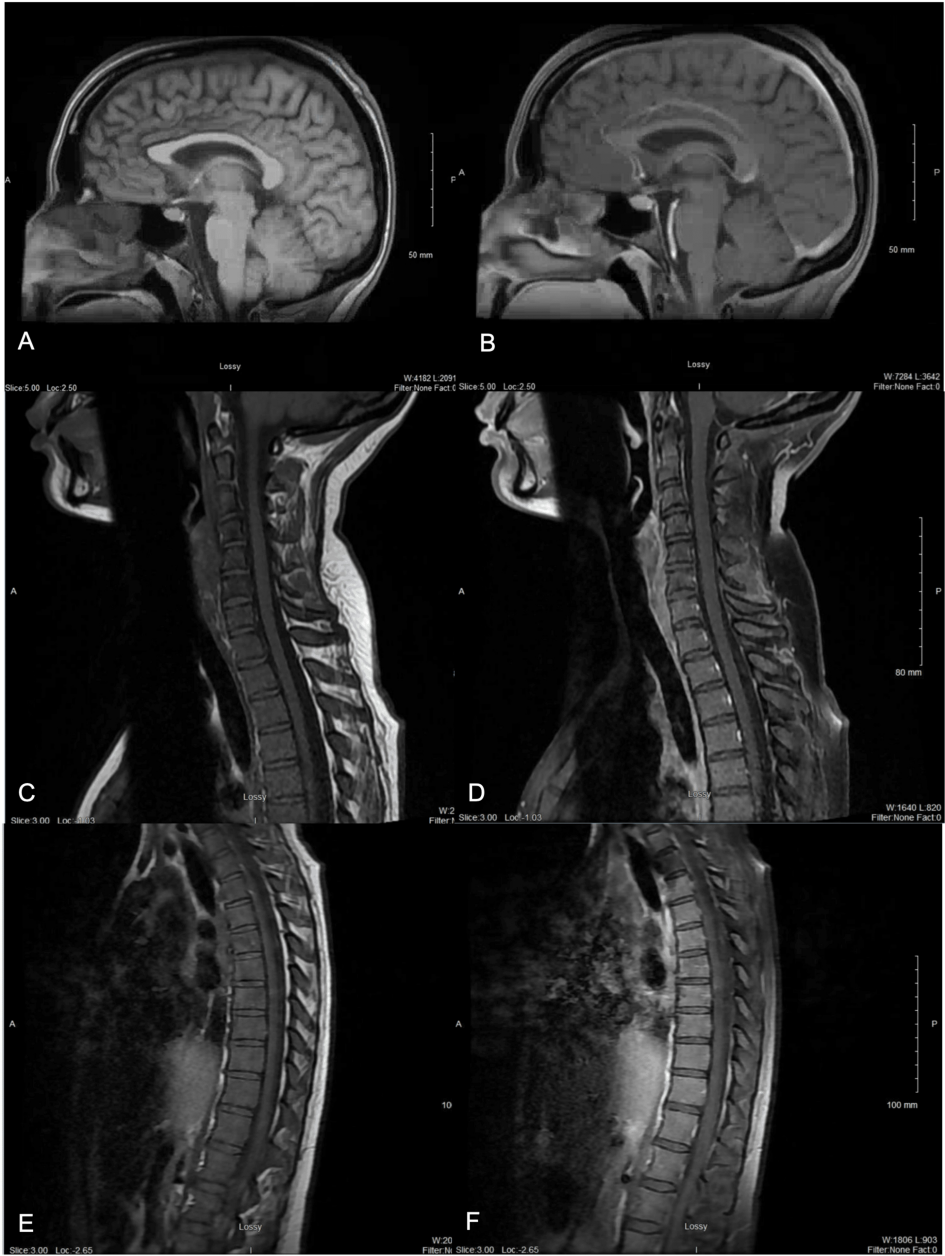
Initial neuraxial MRI with and without contrast, sagittal view. MRI taken at symptom onset. No acute findings noted. (A) Brain MRI without contrast; (B) brain MRI with contrast; (C) cervical MRI without contrast; (D) cervical MRI with contrast; (E) thoracic MRI without contrast; (F) thoracic MRI with contrast.

In the ED, she was evaluated by the neurology consult team. Physical examination revealed a sensory level just below the knees bilaterally, four-plus bilateral patellar reflexes, hyperalgesia, bilateral clonus, bilateral positive Babinski sign, and bilateral foot drop. Sensory testing showed diminished pain, vibration, proprioception, and light touch below the knees bilaterally. Motor strength was decreased (3/5) in bilateral lower extremities and preserved (5/5) in upper extremities. Cranial nerves were intact. No dysmetria or intention tremor was noted. Upper extremity reflexes were normal, and there were no signs of meningeal irritation. Coordination and speech were normal. Upper motor neuron signs on exam raised concern for a central process, and urgent MRI of the brain and entire spine with and without contrast was obtained, which was negative for any cord lesions (Figure 1).

Given the high clinical suspicion for transverse myelitis despite the negative MRI, the patient was admitted to the inpatient neurology service and initiated on high-dose intravenous methylprednisolone (one g/day for five days). CSF analysis was then performed and revealed elevated gamma globulin levels with normal protein electrophoresis, consistent with a nonspecific polyclonal immune response. Additional findings included positive myelin basic protein and evidence of prior Epstein-Barr virus (EBV) exposure. Upon culmination of steroid treatment, the patient was discharged from the hospital. However, she demonstrated minimal improvement and persistent disability. Nerve conduction studies (NCS) and electromyography (EMG) done in the outpatient setting were consistent with a central nervous system process, showing preserved sural sensory potentials and no signs of demyelination. EMG revealed increased amplitude and duration of motor unit potentials (MUPs) with a fixed firing pattern and no signs of peripheral regeneration, supporting central rather than peripheral pathology.

**Figure 2:**
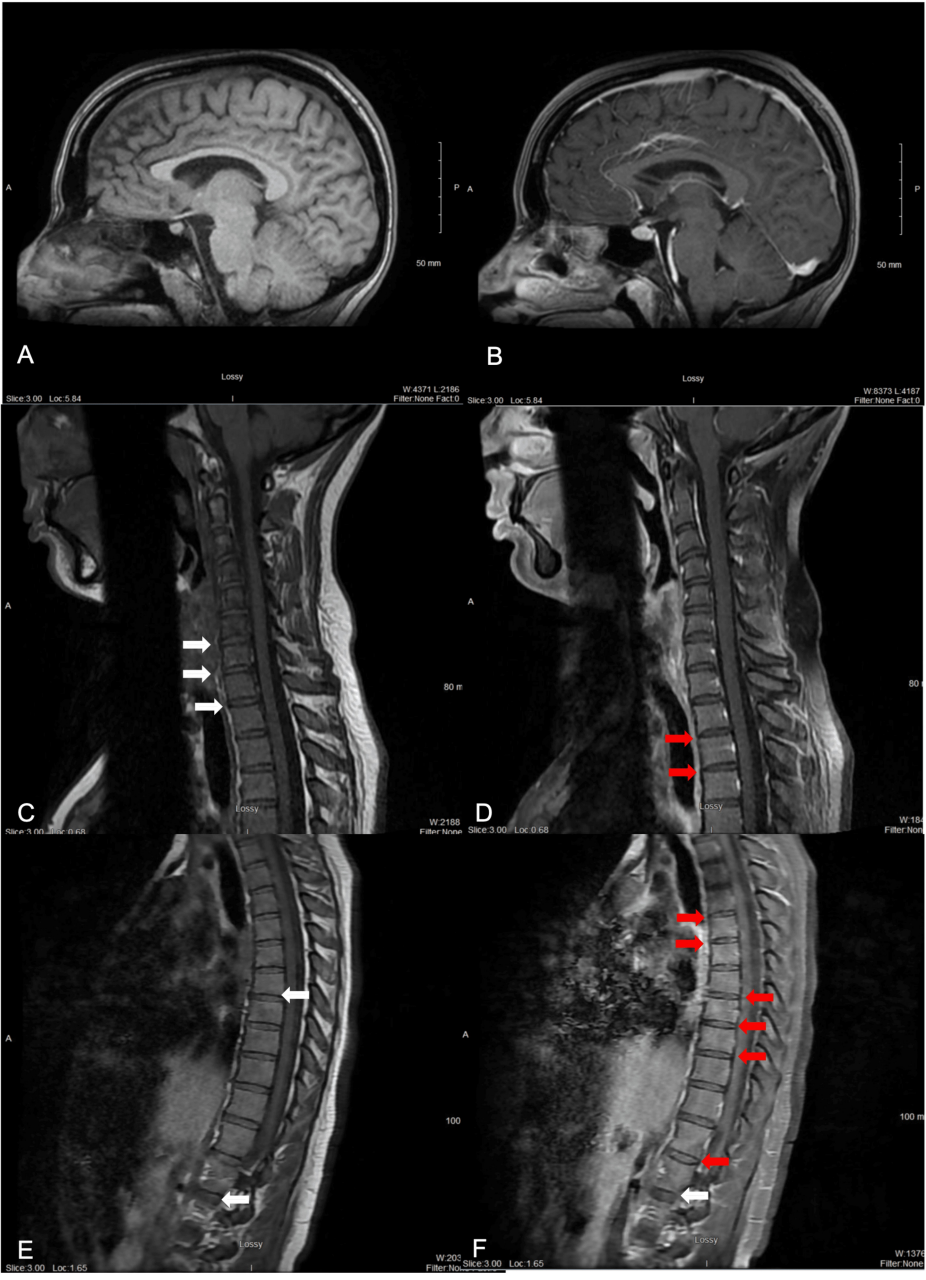
Repeat neuraxial MRI with and without contrast, sagittal view. Image taken three months after symptom onset. (A) Brain MRI without contrast; (B) brain MRI with contrast; (C) cervical MRI without contrast; (D) cervical MRI with contrast; (E) thoracic MRI without contrast; (F) thoracic MRI with contrast. White arrows: disc dehydration. Red arrows: possible bamboo spine.

Due to minimal response to steroids, persistent disability, and evidence of a central process on EMG and NCS, the patient was re-admitted to the inpatient neurology service to start intravenous immunoglobulin (IVIG) therapy for five days. Following this first round of treatment, the patient reported persistent but improved sensory loss to light touch, vibration, proprioception, and pain, which became manageable with pregabalin and physical therapy. Sensory level was now noticed two inches above the bilateral ankles. Babinski signs were still. Patellar reflexes were 3+ bilaterally, and no clonus was noticed. Throughout the first month following the first round of IVIG, symptoms slowly worsened but did not reach the severity experienced at the time of onset. The patient was re-admitted to the inpatient neurology service to receive a second round of 5 days of IVIG. Similarly, she reported symptomatic improvement following treatment. Repeat outpatient NCS/EMG showed resolution of the previously observed central fixed firing pattern, indicating improvement in motor unit recruitment and supporting clinical recovery. However, EDSS score remained at >5, and repeat neuraxial MRI showed disc dehydration and potential bamboo spine, suggesting an underlying degenerative spinal condition (Figure 2).

Given the patient’s symptoms, family history of autoimmune disease, and MRI findings suggestive of spondyloarthropathy, she was evaluated by an outpatient rheumatologist. Extensive testing revealed a negative human leukocyte antigen B27 (HLA-B27), a positive Rheumatoid Factor (RF), and negative results for additional autoimmune markers (Table 1). Although she had no joint swelling, previously reported joint stiffness and imaging findings supported the diagnosis of seropositive RA with extra-articular neurologic involvement. The patient began monthly IVIG treatments and physical therapy. After rheumatologic evaluation and shared decision-making, she was started on certolizumab pegol (Cimzia, UCB Pharma, Parsippany, NJ). Her rheumatologic and neurologic symptoms were closely monitored during outpatient follow-up.

**Table 1:** Comprehensive autoimmune and paraneoplastic antibody panel results. All listed autoantibodies and related immunologic tests were performed to evaluate for underlying autoimmune, paraneoplastic, or neuroinflammatory etiologies. *Negative* results indicate no detectable antibody presence. Values within the reference range are considered normal. IgG and complement levels assess immune system status. Immunofixation evaluates for monoclonal proteins. CCP IgG/IgA Ab, cyclic citrullinated peptide immunoglobulin G/immunoglobulin A antibody; ANA, antinuclear antibody; c-ANCA, cytoplasmic antineutrophil cytoplasmic antibody; PR3 Ab, proteinase 3 antibody; p-ANCA, perinuclear antineutrophil cytoplasmic antibody; MPO, anti-myeloperoxidase; Scl-70 Ab, scleroderma antibody; LGI1 IgG Ab (CBA), leucine-rich glioma-inactivated 1 immunoglobulin G antibody; AGNA-1, anti-glial nuclear antibody type 1; ITPR1 (CBA), inositol 1,4,5-triphosphate receptor type 1; Anti-Yo Ab, anti-Yo antibody; PCA-2, Purkinje cell antibody type 2; PCA-Tr DNER Ab (IFA), Purkinje cell antibody-Tr delta and notch-like epidermal growth factor–related antibody; CRMP-5 IgG Ab, collapsin response mediator protein 5 immunoglobulin G antibody; β-2-GPI IgG Ab, beta-2 glycoprotein I immunoglobulin G antibody; β-2-GPI IgA Ab, beta-2 glycoprotein I immunoglobulin A antibody; β-2-GPI IgM Ab, beta-2 glycoprotein I immunoglobulin M antibody; NMO/AQP-4 IgG Ab, neuromyelitis optica/aquaporin-4 immunoglobulin G antibody; MOG-IgG Ab, myelin oligodendrocyte glycoprotein immunoglobulin G antibody; anti-IgLON5 antibody, anti–immunoglobulin-like cell adhesion molecule 5 antibody; anti-cardiolipin IgG Ab, anti-cardiolipin immunoglobulin G antibody; anti-cardiolipin IgA Ab, anti-cardiolipin immunoglobulin A antibody; anti-cardiolipin IgM Ab, anti-cardiolipin immunoglobulin M antibody; NMDA receptor Ab, N-methyl-D-aspartate receptor antibody; AMPA receptor 1 Ab, alpha-amino-3-hydroxy-5-methyl-4-isoxazolepropionic acid receptor 1 antibody

Test	Result	Reference range/expected
Autoimmune interpretation	Negative	Negative
IgG	1,290 mg/dL	586-1,602 mg/dL
Urine immunofixation	Apparent normal immunofixation pattern	-
CCP IgG/IgA Ab	11 units	0-19 units
ANA confirmation	Negative	-
c-ANCA antibody	<1:20	Neg < 1:20 titer
Proteinase 3 (PR3) Ab	<0.2	0.0-0.9 units
Atypical p-ANCA	<1:20	Neg < 1:20 titer
p-ANCA antibody	<1:20	Neg < 1:20 titer
Anti-myeloperoxidase	<0.2	0.0-0.9 units
Scl-70 (scleroderma) Ab	0.2	0.0-0.9 AI
LGI1 IgG Ab (CBA)	Negative	Negative
Anti-Ma2/Ta Ab	Negative	Negative
Anti-Zic4 antibody	Negative	Negative
Neuronal nuclear Ab type	Negative	Negative
AGNA-1	Negative	Negative
ITPR1 (CBA)	Negative	Negative
Anti-Hu antibody	Negative	Negative
Anti-Ri IgG Ab (IB)	Negative	Negative
Anti-Yo antibody	Negative	Negative
Purkinje cell (PCA-2)	Negative	Negative
Purkinje cytoplasmic type	Negative	Negative
PCA-Tr DNER Ab (IFA)	Negative	Negative
CRMP-5 IgG antibody	Negative	Negative
Amphiphysin antibody	Negative	Negative
β-2-GPI IgG Ab	<9	0-20
β-2-GPI IgA Ab	<9	0-25
β-2-GPI IgM Ab	<9	0-32
NMO/AQP-4 IgG Ab	<1.5	0.0-3.0 U/mL
MOG-IgG Ab	Negative	Negative
Thyroglobulin antibody	<1.0	0.0-0.9 IU/mL
Anti-IgLON5 antibody	Negative	Negative
Anti-cardiolipin IgG Ab	<9	0-14 GPL U/mL
Anti-cardiolipin IgA Ab	<9	0-11 APL U/mL
Anti-cardiolipin IgM Ab	<9	0-12 MPL U/mL
NMDA receptor Ab	Negative	Negative
AMPA receptor 1 Ab	Negative	Negative
AMPA receptor 2 Ab	Negative	Negative
Complement C4	23 mg/dL	12-38 mg/dL

## Discussion

This case is significant for a TM-like presentation in a patient without a prior autoimmune diagnosis, who was diagnosed with seropositive rheumatoid arthritis based on clinical features, positive RF, and supportive imaging findings. Degenerative spinal changes with no cord compression further supported an underlying rheumatologic condition. Alternative causes of myelopathy were thoroughly evaluated. Guillain-Barré syndrome was unlikely given the presence of upper motor neuron signs, brisk reflexes, and central findings on EMG/NCS. Compressive myelopathy was excluded by repeated negative MRI imaging of the neuraxis. Multiple sclerosis and NMOSD were considered but deemed unlikely based on clinical presentation, absence of typical imaging features, and negative neuromyelitis optica/aquaporin-4 (NMO/AQP-4 IgG) antibodies. Spinal cord infarction was also considered; however, the subacute progression and CSF findings supported an inflammatory rather than ischemic etiology. Extensive laboratory evaluation further excluded alternative autoimmune, paraneoplastic, and inflammatory etiologies. Negative autoimmune encephalitis panel antibodies, including NMDA receptor, AMPA receptors 1 and 2, LGI1, anti-Ma2/Ta, anti-Hu, anti-Ri, anti-Yo, and anti-Zic4, ruled out paraneoplastic or autoimmune encephalitic syndromes. Additionally, the absence of anti-nuclear antibodies (ANA), negative scleroderma-associated antibodies (Scl-70), negative antiphospholipid and cardiolipin antibodies, and normal complement C4 levels effectively excluded other systemic autoimmune and complement-mediated inflammatory diseases. Collectively, these results robustly confirmed the diagnosis of MRI-negative transverse myelitis associated with seropositive RA.

A prior study examining myelitis in the context of rheumatologic diseases identified a case of rheumatoid arthritis among the affected patients [11]. Seropositive RA is associated with a more severe disease course, a greater risk of extra-articular manifestations, and a higher degree of systemic immune dysregulation compared to seronegative RA. Studies suggest that, in seropositive RA, the earliest pathological events occur at extra-articular sites, where autoreactive T cells and autoantibodies expand well before clinical arthritis becomes apparent [12]. These systemic features may explain the neurological involvement seen in our patient. Notably, unlike most TM cases that demonstrate T2-hyperintense lesions on imaging, our patient’s MRI remained negative throughout. This highlights a diagnostic gap in imaging-based algorithms and the importance of considering other MRI-negative etiologies that may mimic TM, such as MOG-associated disease, NMOSD, paraneoplastic syndromes, and sarcoidosis [13]. This case highlights the importance of maintaining a high index of suspicion for autoimmune myelitis, even in the absence of imaging findings, particularly when supported by a clinical examination and CSF analysis. It emphasizes the need to consider the presence of rheumatologic conditions, such as RA, when building a differential diagnosis that includes transverse myelitis. Early recognition and initiation of immunotherapy, such as IVIG, can lead to partial functional recovery [14]. In our patient, IVIG resulted in improved sensory symptoms and reduced pain, although significant lower extremity weakness and mobility limitations persisted.

In this patient, the development of TM may be a result of genetic autoimmune susceptibility and environmental immune triggering. Autoimmune rheumatic diseases like RA arise from genetic predisposition and environmental risk factors. Infections can trigger or exacerbate autoimmune conditions through well-established mechanisms, including molecular mimicry, epitope spreading, bystander activation, and exposure to cryptic antigens. Molecular mimicry occurs when pathogens and host tissues share similar peptide sequences, leading to cross-reactive immune responses. Epitope spreading can misdirect immune surveillance from infectious to self-antigens. Bystander activation occurs when inflammation indiscriminately activates autoreactive T cells, while cryptic antigens may be revealed during tissue injury. These pathways are implicated in diseases such as SLE and MS and may also contribute to the pathogenesis of autoimmune myelitis. The recent skin infection in our patient might have triggered these mechanisms, setting off an immune cascade that targeted the spinal cord [15,16].

The use of corticosteroids is the first-line treatment for several common autoimmune neurologic conditions; however, in this case, the patient showed minimal response to high-dose steroids, requiring escalation to IVIG as an alternative therapeutic approach. IVIG is a second-line therapy used in steroid-refractory autoimmune neurologic conditions. While its exact mechanism is not fully understood, it modulates the immune response and has shown clinical benefit in such cases [17]. Following rheumatologic evaluation, the patient was started on certolizumab pegol (Cimzia), a subcutaneous tumor necrosis factor-alpha (TNFα) inhibitor. This decision was based on her presentation, serologic findings, previously reported joint stiffness, and imaging suggestive of underlying inflammatory arthropathy. Clinical studies have demonstrated that Cimzia improves pain and functional outcomes, as measured by the American College of Rheumatology 50% (ACR50) response rate compared to placebo [18]. Although studies have reported a potential association between TNFα inhibitors and inflammatory CNS events, the evidence remains limited, and no definitive pathogenic mechanism has been identified. As such, the clinical significance of this association is uncertain and must be interpreted with caution [19]. This case describes an MRI-negative TM secondary to seropositive RA. Cimzia was started on a shared decision basis, weighing risks and benefits. Close neurologic monitoring will be essential in this case, and continuation of IVIG is expected due to the significant clinical improvement observed.

## Conclusions

In conclusion, this case reinforces the understanding that transverse myelitis, though rare, can occur as a neurologic manifestation of seropositive rheumatoid arthritis, including in the absence of radiographic findings. It demonstrates that transverse myelitis can occur as a presenting symptom of rheumatoid arthritis. It underscores the importance of clinical vigilance, thorough autoimmune workup, and immunotherapy in patients with suspected autoimmune myelitis, even when MRI findings are absent. Further investigation is needed to characterize MRI-negative TM better and to understand the mechanisms that link systemic autoimmune disease to central nervous system inflammation.
